# The Effect of Y/Er and Zn Addition on the Microstructure and Mechanical Properties of Mg-11Li Alloy

**DOI:** 10.3390/ma12193066

**Published:** 2019-09-20

**Authors:** Mingquan Zhang, Jinghuai Zhang, Ruizhi Wu, Hongwei Cui, Ertuan Zhao, Shujuan Liu, Pengfei Qin, Qing Ji

**Affiliations:** 1 Key Laboratory of Superlight Material and Surface Technology, Ministry of Education, College of Material Science and Chemical Engineering, Harbin Engineering University, Harbin 150001, China; zmq1106435777@163.com (M.Z.); rzwu@hrbeu.edu.cn (R.W.); pfqin@ciac.ac.cn (P.Q.); jiqing@hrbeu.edu.cn (Q.J.); 2 College of Materials Science and Engineering, Shandong University of Technology, Zibo 255000, China; chw@sdut.edu.cn; 3 School of Mechanical Engineering, Shandong University of Technology, Zibo 255000, China; etzhao@sdut.edu.cn; 4 Department of Materials Physics and Chemistry, Harbin Institute of Technology, Harbin 150001, China; liusj0817@hit.edu.cn

**Keywords:** Mg-Li alloy, rare earth, microstructure, mechanical properties

## Abstract

Although body-centered cubic (BCC) structural magnesium–lithium (Mg-Li) alloys have lower density and better formability than common hexagonal close-packed (HCP) Mg alloys, their applications remain limited due to their low strength. The purpose of this study is to investigate the effect of Y/Er and Zn addition on the microstructure and tensile properties of Mg-11Li alloy with a BCC structural matrix by comparing Mg-11Li, Mg-11Li-4Y-2Er-2Zn, and Mg-11Li-8Y-4Er-4Zn (wt %) alloys. The results indicate that the addition of Y/Er and Zn at a ratio of 3:1 cannot promote the formation of long-period stacking ordered structure in Mg-11Li alloy such as that in Mg-Y-Er-Zn alloys and the dominant intermetallic phases formed are BCC Mg_24_RE_5_ and face-centered cubic (FCC) Mg_3_RE_2_Zn_3_ phases. With an increase of the content of Y/Er and Zn in an as-cast alloy, the fraction of intermetallic particles increases and the grain size decreases. The addition of Y/Er, as well as Zn, dramatically promotes the refinement of dynamic recrystallization (DRX) during extrusion. The initial intermetallic phases induced by Y/Er and Zn addition are broken into relatively fine particles during extrusion, and this contributes to refining the dynamic recrystallized (DRXed) grains mainly by the particle stimulated nucleation mechanism. The as-extruded Mg-11Li-4Y-2Er-2Zn and Mg-11Li-8Y-4Er-4Zn alloys exhibit much higher tensile strength as compared with as-extruded Mg-11Li alloy, which is mainly ascribed to the refined DRXed grains and numerous dispersed intermetallic phase particles. It is suggested that further refinement of intermetallic particles in these extruded Mg-11Li-based alloys may lead to higher quality alloy materials with low density and excellent mechanical properties.

## 1. Introduction

Magnesium (Mg) alloys, as lightweight structural metallic materials, have great potential in automotive and aerospace applications [1,2,3,4], however, some inherent weaknesses, such as poor formability at room temperature owing to the limited slip systems in the hexagonal close-packed (HCP) structure, restrict their applications [5,6]. To overcome this drawback, lithium (Li) has been used to change the crystal structure of Mg alloys. Specifically, the crystal structure of Mg-Li alloys change from an α (HCP) structure of Mg solid solution to a β (BCC) structure of Li solid solution when the amount of the Li added in Mg exceeds ~11 wt % [7,8], which can significantly improve the formability by providing more slip systems at room temperature [9,10,11,12]. In addition, although high Li content improves the formability and further reduces the density of Mg alloys, it greatly reduces the strength of Mg alloys, and the ultimate tensile strength (UTS) of common Mg-Li alloys containing high Li content is rarely more than 200 MPa even after severe plastic deformation (equal channel angular extrusion or hot extrusion) [13,14,15,16], which is also not conducive to the wide application of Mg alloys.

At present, it is well-known that the addition of rare earth (RE) can effectively enhance the strength and heat resistance of Mg alloys, thus forming typical Mg–RE series and Mg–RE–Zn series alloys [17,18,19,20,21]. Our latest research results show that Mg-Y-Er-Zn extruded alloys have excellent strength both at room and high temperatures mainly due to the formation of a long-period stacking ordered (LPSO) structure or stacking faults [22,23]. Moreover, the influence of RE combined with Zn on some Mg-Li alloys has also been studied and good results have been obtained. Our previous study [24] found that adding Y and Zn (Y/Zn = ~3) to Mg-8Li (wt %) alloy can form a LPSO phase, and thus greatly improve the mechanical properties. Xu et al. [25] added Zn and Y elements (Zn/Y = ~5) simultaneously into Mg-6Li (wt %) alloy, and the results showed that the strength of Mg-6Li alloy was improved by forming a quasicrystal phase. Zhu et al. [26] revealed that the combined addition of Y and Nd improved the tensile properties of Mg-5Li-3Al-2Zn (wt %) alloy mainly due to the grain refinement strengthening mechanism. Zhang et al. [27] reported that the enhanced strength of the extruded Mg-9Li-6Zn-2Gd (wt %) alloy was ascribed to the grain refinement and the formation of a quasicrystal phase. Our comprehensive literature investigation indicates that most of the research has been focused on the effect of RE combined with Zn on single (α phase) or binary (α+β phase) matrix phase Mg-Li alloys with a relatively low Li content.

On the basis of our previous research about Mg-Li-based and Mg-Y-Er-Zn alloys, in this work, we carried out a comparative study on the β-structure Mg-11Li (wt %) alloy with and without RE (Er and Y) and Zn additions. The benchmark alloy was chosen as Mg–11Li (wt %) alloy and compared with the designed Mg-11Li-4Y-2Er-2Zn (wt %) and Mg-11Li-8Y-4Er-4Zn (wt %) alloys. (i.e., the amount of RE is 6%, 12%, and RE:Zn = 3:1). The aim of this paper was to understand the effect of such addition of Y/Er and Zn on the microstructure and mechanical properties of Mg-11Li alloy.

## 2. Experimental Procedures

The cast ingots with designed compositions of Mg-11Li (wt %), Mg-11Li-4Y-2Er-2Zn (wt %), and Mg-11Li-8Y-4Er-4Zn (wt.%) were prepared by melting commercial pure Mg (99.9 wt %), Li (99.9 wt %), Zn (99.9 wt %), Mg-20Y (wt %), and Mg-20Er (wt %) master alloys in a vacuum medium frequency electromagnetic induction furnace at 993 K for 30 min under the protection of argon. The melt was poured into a permanent mold with a diameter of approximately 90 mm at ~973 K. The chemical composition of the obtained ingot was examined using an inductively coupled plasma analyzer (ICP) (Optima 8000DV, perkinElmer, Waltham, MA, USA) and the results are listed in Table 1. Before extrusion, the ingots were processed into billets with a diameter of 80 mm. After preheating at 373 K for 2 h, the billet was extruded into the bars with a diameter of 20 mm at the same temperature under a ram speed of 0.1 mm/s and an extrusion ratio of 16.

The microstructure was characterized using an optical microscope (OM) (ZEISS Axiovert200 MAT, Carl-Zeiss Co., Yarra, Germany), a scanning electron microscope (SEM) (Merlin Compact, Carl Zeiss, Jena, Germany) with an accelerating voltage of 20 KV and equipped with an X-ray energy-dispersive spectrometer (EDS) (Oxford EDS, Oxford Instruments, Oxford, UK), a transmission electron microscope (TEM) (Tecnai G20, FEI, Hillsboro, OR, USA) operated at 200 KV, and an X-ray diffractometer (XRD) (Bruker D8 Advance, Bruker AXS, Karlsruhe, Germany). The polished samples for OM and SEM characterizations were etched by 3% nitric acid alcohol solution. Thin foils with a 3 mm diameter for TEM observation were prepared by the argon ion thinning technique. 

The tensile specimens were cut from the extruded bars, and the tensile direction was parallel to the extrusion direction (ED). Tensile tests were performed using a testing machine (Instron 5869, Norwood, MA, USA) with an initial strain rate of 1 × 10^−3^ s^−1^ at room temperature. There were at least four test bars for each tensile test and the tensile data were an average value of the tensile specimens. 

## 3. Results

### 3.1. Microstructure of the As-Cast Alloys

**Figure 1:**
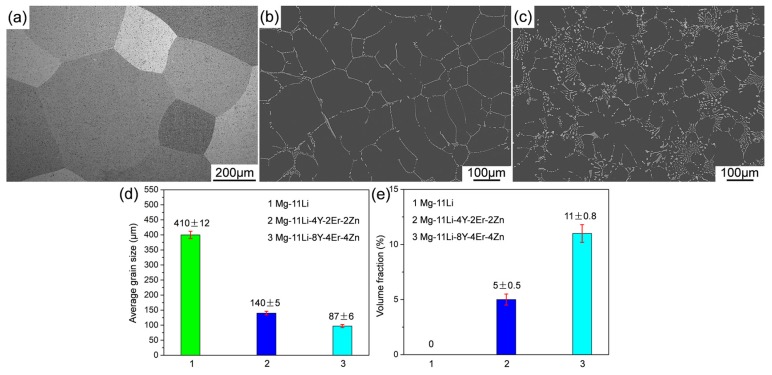
Microstructure of the as-cast alloys: (**a**) optical microscope (OM) image of Mg-11Li, scanning electron microscope (SEM) images of (**b**) Mg-11Li-4Y-2Er-2Zn and (**c**) Mg-11Li-8Y-4Er-4Zn, (**d**) average grain size, and (**e**) average volume fraction of secondary phases.

Figure 1 shows the OM and SEM images of the as-cast Mg-11Li, Mg-11Li-4Y-2Er-2Zn, and Mg-11Li-8Y-4Er-4Zn alloys. The Mg-11Li alloy exhibits a distinct single-phase structure with large grain size and no intermetallic particles are observed (Figure 1a). After 6RE and 2Zn addition, the intermetallic particles appear and are continuously distributed along grain boundaries (GBs) (Figure 1b). With a further increase of RE and Zn content, much more intermetallic particles are formed along GBs and they had the tendency to disperse into the grains (Figure 1c). Their average grain sizes, measured by linear intercept method, are presented in Figure 1d. It can be seen clearly that with the addition of RE and Zn, the grain size of Mg-11Li-based alloys significantly decreases, indicating a satisfactory grain refinement effect. Figure 1e presents the statistic, volume fraction of intermetallic phase versus RE and Zn content. Obviously, the volume fraction of the intermetallic phase increases monotonously as RE and Zn content increases.

**Figure 2:**
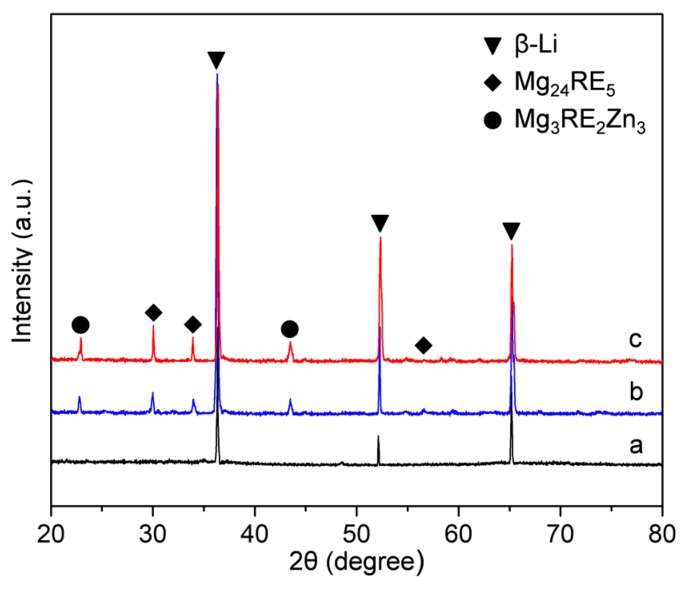
The X-ray diffractometer (XRD) patterns of the as-cast alloys: (**a**) Mg-11Li, (**b**) Mg-11Li-4Y-2Er-2Zn, and (**c**) Mg-11Li-8Y-4Er-4Zn.

Figure 2 shows the XRD patterns obtained from the three as-cast alloys. For the Mg-11Li alloy, there are no obvious additional diffraction peaks, except those from the β-Li phase, indicating no other phase in Mg-11Li alloy, which is consistent with OM observation. After the addition of 6Y/Er and 2Zn to Mg-11Li alloy, the extra diffraction peaks appear, which are close to the standard diffraction peaks of Mg_24_Y_5_/Mg_24_Er_5_ and Mg_3_Y_2_Zn_3_/Mg_3_Er_2_Zn_3_ (namely W phase in other literature)_._ As the RE and Zn content increases, the intensity of diffraction peak of Mg_24_RE_5_ and W phases increase and no other diffraction peaks appear, which indicates that the volume fraction of the intermetallic phases, Mg_24_RE_5_ and W, increases and no obvious other intermetallic phase forms in the Mg-11Li-based alloy.

**Figure 3:**
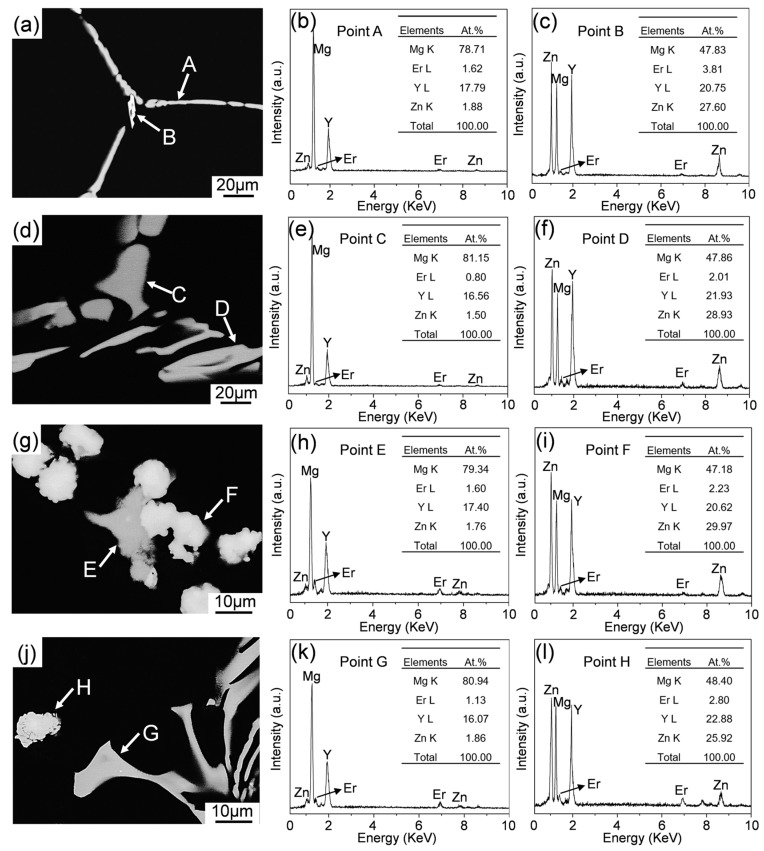
Backscattered electron SEM (BSE-SEM) images and corresponding energy-dispersive spectrometer (EDS) results of the typical intermetallic phases in as-cast alloys: (**a**–**f**) Mg-11Li-4Y-2Er-2Zn and (**g**–**l**) Mg-11Li-8Y-4Er-4Zn.

Figure 3 shows the backscattered electron SEM (BSE-SEM) images of typical intermetallic phases along GBs and corresponding EDS results of as-cast Mg-11Li-4Y-2Er-2Zn and Mg-11Li-8Y-4Er-4Zn alloys. Under the BSE-SEM mode (Figure 3a,d), it can be found by careful observation that the secondary phases in Mg-11Li-4Y-2Er-2Zn alloy can be divided into two types according to their brightness and morphology. One presents roughly continuous rod-like morphology (A and C), and the other is brighter with discontinuous irregular block morphology (B and D). Combined with the analysis result of XRD, the EDS results show that the roughly continuous rods are Mg_24_(Y,Er)_5_, in which RE is dominated by Y and there is also a small amount of dissolved Zn, and the discontinuous irregular blocks with higher brightness are identified as W phase Mg_3_(Y,Er)_2_Zn_3_, where RE is also dominated by Y. As for the low content of Er in intermetallic phases, this could be related to the high solid solubility and low alloying content of Er. As the RE and Zn content increases, the two dominant types of intermetallic phases in Mg-11Li-based alloy are not changed based on the EDS results, while under BSE-SEM observation, their morphologies change obviously, that is, the Mg_24_(Y,Er)_5_ phase is thicker (E and G), while the W phase becomes spheroidized and has a rough surface (F and H).

**Figure 4:**
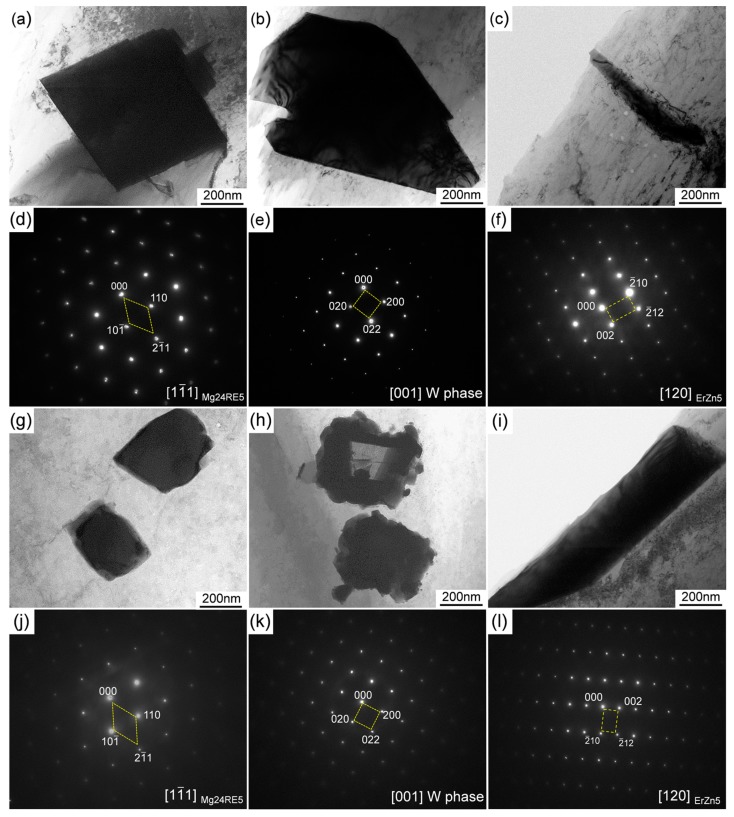
Bright-field transmission electron microscope (BF-TEM) images and corresponding selected area electronic diffraction (SAED) patterns of the intermetallic phases in the as-cast alloys: (**a**–**f**) Mg-11Li-4Y-2Er-2Zn and (**g**–**l**) Mg-11Li-8Y-4Er-4Zn.

Figure 4 shows the typical bright-field TEM (BF-TEM) images and the corresponding selected area electronic diffraction (SAED) patterns, to detect the intermetallic phases in as-cast alloys which are too small in size to be easily observed by TEM. The TEM analysis indicates that there are three types of intermetallic phases in as-cast Mg-11Li-4Y-2Er-2Zn alloy. Combined with the XRD analysis, the quadrate particle (Figure 4a) is identified as Mg_24_RE_5_ (body-centered cubic structure, a = 1.122 nm [28]) by SAED (Figure 4d) and the blocky phase (Figure 4b) is considered to be W phase (face-centered cubic structure, a = 0.683 nm [28]) by SAED as in Figure 4e. Similarly, the quadrate phase (Figure 4g) and globular phase, with coarse surface (Figure 4h) in as-cast Mg-11Li-8Y-4Er-4Zn alloy, are identified by the corresponding SAED patterns to be Mg_24_RE_5_ and W phase, respectively (Figure 4j,k). The above analyses show the consistency between TEM and XRD, as well as SEM. In addition, a new stick-like phase (Figure 4c,i) is also found by TEM in both of the as-cast alloys. On the basis of the alloy composition and SAED analysis, this stick-like phase is speculated to be an ErZn_5_ phase (close-packed hexagonal structure, a = 0.884 nm and c = 0.918 nm [29]) and it cannot be identified by XRD, indicating it’s low content in the alloys.

### 3.2. Microstructure of As-Extruded Alloys

**Figure 5:**
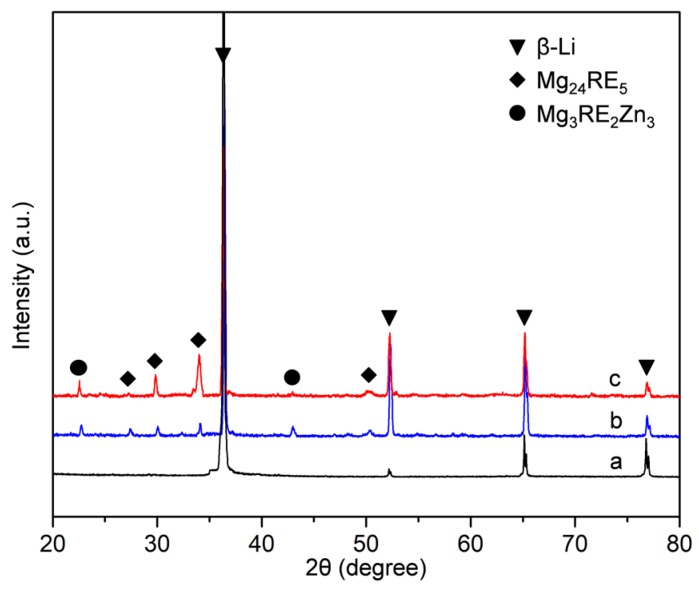
XRD patterns of the as-extruded alloys: (**a**) Mg-11Li, (**b**) Mg-11Li-4Y-2Er-2Zn, and (**c**) Mg-11Li-8Y-4Er-4Zn.

**Figure 6:**
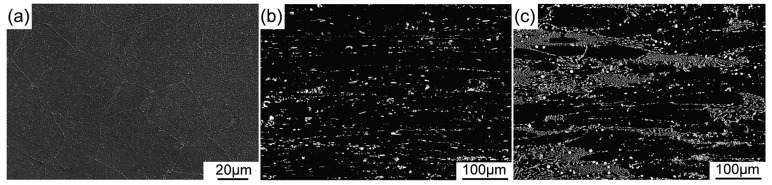
SEM images of the as-extruded alloys: (**a**) Mg-11Li, (**b**) Mg-11Li-4Y-2Er-2Zn, and (**c**) Mg-11Li-8Y-4Er-4Zn.

**Figure 7:**
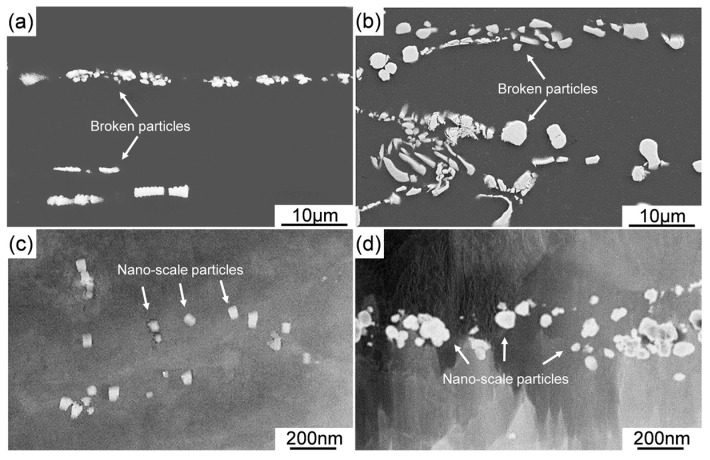
Magnified SEM and high-angle annular dark field scanning transmission electron microscopy (HAADF-STEM) images of the particles in as-extruded alloys: (**a** and **c**) Mg-11Li-4Y-2Er-2Zn and (**b** and **d**) Mg-11Li-8Y-4Er-4Zn.

Figure 5 presents the XRD patterns of as-extruded Mg-11Li, Mg-11Li-4Y-2Er-2Zn, and Mg-11Li-8Y-4Er-4Zn alloys, indicating that the dominant phases are unaltered as compared with the corresponding as-cast alloys. Figure 6 shows the SEM images of the three alloys after extrusion. The Mg-11Li alloy consists of the equiaxed dynamic recrystallization (DRX) grains and the average size of grains is ~40 μm (Figure 6a). Compared with the grain size (410 μm) of as-cast Mg-11Li alloy, its grain size is significantly refined by extrusion at 373 K, but the grain size of 40 μm is still too large for the extruded Mg alloy. This is mainly because the recrystallization temperature of Mg-11Li alloy with high Li content is low, which leads to easy recrystallization and growth during extrusion. After Y/Er and Zn addition, it can be found in as-extruded Mg-11Li-4Y-2Er-2Zn alloy that numerous intermetallic particles are crushed by extrusion and roughly dispersed along the ED (Figure 6b). As the Y/Er and Zn content increases, more broken particles form the particle bands distributed along the ED in as-extruded Mg-11Li-8Y-4Er-4Zn alloy (Figure 6c). The magnified SEM images indicate that the size of the crushed intermetallic particles ranged from about 1 to 5 μm in as-extruded Mg-11Li-4Y-2Er-2Zn (Figure 7a) and Mg-11Li-8Y-4Er-4Zn (Figure 7b) alloys. Moreover, according to the observation by high-angle annular dark field scanning transmission electron microscopy (HAADF-STEM), a large number of nanoscale particles with a size ranging from 20–100 nm are formed in both studied alloys (Figure 7c,d). Unfortunately, the grain structure of Mg-11Li-based alloys is not observed under SEM as well as HAADF-STEM after Y/Er and Zn addition.

**Figure 8:**
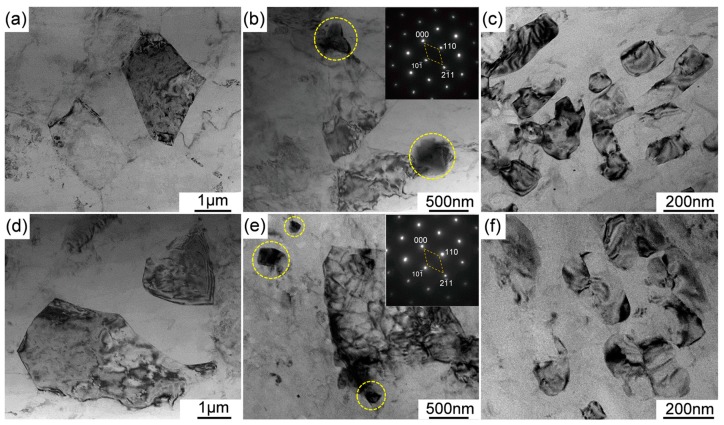
BF-TEM images to showing the recrystallized (DRXed) grains in as-extruded alloys: (**a**–**c**) Mg-11Li-4Y-2Er-2Zn and (**d**–**f**) Mg-11Li-8Y-4Er-4Zn.

Figure 8 shows the representative BF-TEM images which reveal the recrystallized (DRXed) grains in as-extruded Mg-11Li-based alloys containing RE and Zn. The fine DRXed grains with a size of approximately 1 to 3 μm are formed in Mg-11Li-4Y-2Er-2Zn alloy during extrusion (Figure 8a,b), whereas the RE and Zn content increases and the size of the DRXed grains slightly increase, with the size ranging from 2 to 5 μm (Figure 8d,e). It is worth noting that some nanometer-sized grains with a size of about 100 to 300 nm are also found in both studied alloys (Figure 8c,f). Moreover, some nanoscale particles (yellow dotted circle) are observed around some DRX GBs (Figure 8b,e). According to the corresponding SAED patterns, these particles are confirmed as Mg_24_RE_5_ phase.

### 3.3. Mechanical Properties

**Figure 9:**
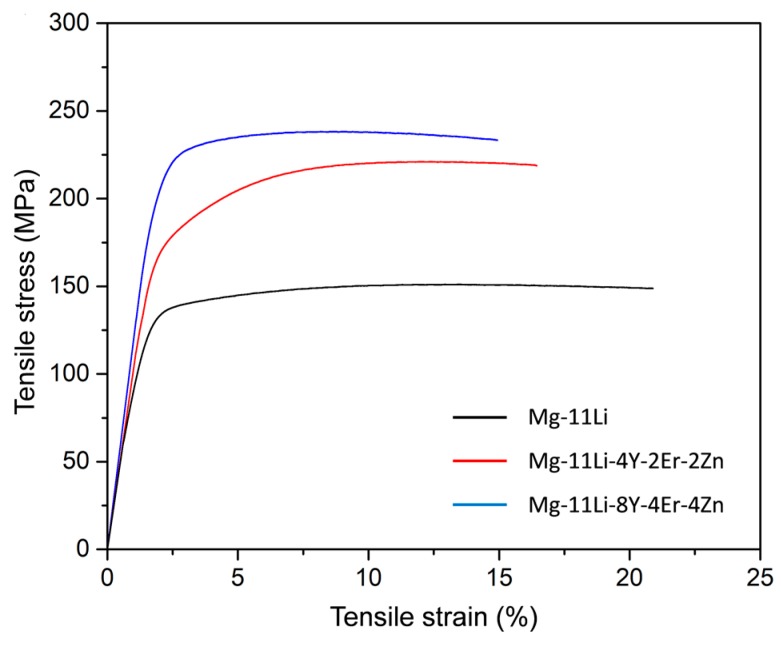
Tensile stress–strain curves of the as-extruded Mg-11Li-based alloys.

Figure 9 shows the typical tensile stress–strain curves for the as-extruded alloys. The average tensile properties including UTS, yield strength (YS) and elongation to failure (*ε*) are listed in Table 2. For the as-extruded Mg-11Li alloy, the UTS, YS, and *ε* are 145 MPa, 117 MPa, and 22%, respectively, revealing the low strength of Mg-Li binary alloy with high Li content despite good plasticity. After adding 6Y/Er and 2Zn, the UTS (224 MPa) and YS (174 MPa) of as-extruded Mg-11Li-based alloy are significantly increased. Adding more Y/Er (12%) and Zn (4%) further increases the UTS (243 MPa) and YS (210 MPa) of as-extruded Mg-11Li-based alloy. The *ε* of the Mg-11Li-based alloys decreases with the addition of Y/Er and Zn, but still remains high level. In addition, although the density (*ρ*) of the Mg-11Li-based alloys increases with the addition of Y/Er and Zn, it is still significantly lower than that of pure Mg (Table 2).

## 4. Discussion

### 4.1. Effect of Y/Er and Zn Addition on Microstructure of Mg-11Li Alloy

According to the reports so far and our preliminary experiments, the main intermetallic phases in both Mg-4Y-2Er-2Zn and Mg-8Y-4Er-4Zn alloys would be LPSO structural Mg_12_(Y,Er)_1_Zn_1_ or Mg_10_(Y,Er)_1_Zn_1_ phases [22]. However, this study indicates that LPSO structure cannot be formed in Mg-Li-Y-Er-Zn alloys after the addition of 11% Li. This reveals that the formation law of LPSO structure in Mg-RE-Zn alloys reported in the literature [30] is not suitable for this kind of Mg-Li-RE-Zn alloys, and also indicates that the effect of Li on the matrix crystal structure would further seriously affect the formation of LPSO structure. The present study indicates that the main secondary phases formed in both as-cast Mg-11Li-4Y-2Er-2Zn and Mg-11Li-8Y-4Er-4Zn alloys are BCC structural Mg_24_RE_5_ and FCC structural W phases, although their morphologies are slightly different in the two alloys.

The grains of as-cast Mg-11Li-based alloys are obviously refined by the addition of Y/Er and Zn. The growth of grain in matrix is connected with the interfacial energy during solidification [31]. The RE atoms, as well as Zn, own a large difference in radius from Mg and Li atoms, and it means that the addition of Y/Er and Zn to Mg alloys can disrupt the arrangement of the atoms. Numerous atomic vacancies on the solid–liquid interface would be generated to a certain extent during the solidification process [31], which could enhance the interface energy of the alloys and offer favorable nucleation sites. Such a trend makes a positive condition for the grain refinement [32,33]. Simultaneously, since the equilibrium partition coefficient, *K,* of Y/Er and Zn solute elements is far less than one, solute atoms Y/Er, as well as Zn, would be enriched and aggregated at the solid–liquid interface during the solidification process, and such enrichment of solute atoms finally results in preferentially nucleate and formation of Mg_24_RE_5_ and W intermetallic phases at GBs, which subsequently act as effective barriers to inhibit grain growth by limiting grain boundary migration [34,35].

After extrusion, the grain of Mg-11Li binary alloy is refined due to DRX, but the DRXed grain is still coarse, while with the addition of Y/Er, as well as Zn, the DRXed grain is significantly refined. It is considered that this should be mainly related to the following two aspects. On the one hand, as the atomic size of Y/Er is much larger than that of Mg and Li (more than 10% in diameter), the Y/Er in an Mg-Li alloy would have a strong trend toward segregation to GBs or any other defect sites to weaken the size misfit energy in the matrix. It has been reported that this segregation would strongly suppress the dynamic recrystallization (DRX) in Mg-based alloys [36]. On the other hand, particle stimulated nucleation (PSN) during hot extrusion has been reported in Mg alloys [37,38]. Zeng et al. [39] have reported that Mg_24_Y_5_ could serve as heterogeneous nucleation sites for β-Li phase in Mg-9Li alloy based on the edge-to-edge matching model. In the present study, the fine broken intermetallic particles, especially Mg_24_RE_5_, can be regarded as the potential grain refiner for the Mg-Li-Y-Er-Zn alloys during the extrusion process (Figure 8b). In addition to PSN, finer particles in GBs can also influence GB movement (pinning effect) during DRX. Moreover, the fine broken Mg_24_RE_5_ and W particles can generate local inhomogeneity of strain energy and stress concentration by hindering the movement of dislocations, and therefore it can enhance driving force for crystallization. Nevertheless, it can be seen that with the increase of Y/Er and Zn content, the DRXed grain size slightly increases (Figure 8). This may be due to too many intermetallic phases that are not broken enough during the extrusion process in Mg-11Li-8Y-4Er-4Zn alloy (Figure 6c). It was reported that the intermetallic particles with a large size would shelter the matrix when deformation occurs, which reduces the strain accumulation and strain energy of the matrix, and finally lowers the driving force for recrystallization [40].

### 4.2. Effect of Y/Er and Zn Addition on the Mechanical Properties of Mg-11Li Alloy

**Figure 10:**
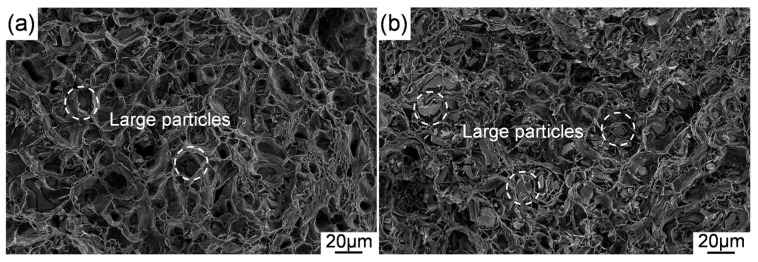
Tensile fracture of as-extruded alloys: (**a**) Mg-11Li-4Y-2Er-2Zn and (**b**) Mg-11Li-8Y-4Er-4Zn.

The present study indicates that the strength of as-extruded Mg-11Li alloy is significantly increased after the addition of Y/Er and Zn. It is considered that the strength improvement is mainly related to the factors as follows: (1) the addition of Y/Er and Zn leads to the formation of numerous intermetallic compounds, Mg_24_(Y,Er)_5_ and Mg_3_(Y,Er)_2_Zn_3,_ in Mg-11Li alloy. The fine broken intermetallic particles caused by extrusion can effectively block the dislocation motion during the tensile testing [38,41], which contributes to the strength improvement via dispersion strengthening. (2) the DRXed grains of as-extruded Mg-11Li alloy are further refined dramatically due to the addition of Y/Er and Zn. The fine DRXed grains contribute to the high strength through grain boundary strengthening based on the Hall–Petch relationship [42]. Simultaneously, grain refinement is also an effective means to improve plasticity. However, the Mg-Li-Y-Er-Zn alloys with finer grains have a lower ε as compared with Mg-11Li binary alloy. The reason for this phenomenon is that there are still relatively coarse intermetallic particles in the Mg-Li-Y-Er-Zn alloys after extrusion in this study. These coarse particles cause stress concentrations at the interface of particle and matrix, resulting in the initiation and propagation of cracks during the deformation. This point can be supported by the fracture observation of Mg-Li-Y-Er-Zn alloys, as shown in Figure 10. It is conceivable that the better mechanical properties of the Mg-Li-Y-Er-Zn alloys can be obtained by further refinement of particles by optimizing the deformation process.

## 5. Conclusions

In this study, the effect of Y/Er and Zn addition on the microstructure and tensile properties of Mg-11Li alloy with BCC structural matrix was investigated by the comparison of Mg-11Li, Mg-11Li--4Y-2Er-2Zn, and Mg-11Li-8Y-4Er-4Zn (wt %) alloys, and the main conclusions are listed as follows:

(1) The addition of Y/Er and Zn at the ratio of 3:1 to Mg-11Li alloy does not promote the formation of LPSO structure such as that in Mg-Y-Er-Zn alloys, and the dominant intermetallic phases formed are BCC Mg_24_RE_5_ and FCC W phases. The fraction of intermetallic phases increases, and the grain size of the as-cast alloys decreases with increasing Y/Er and Zn content.

(2) The effect of Y/Er and Zn added to Mg-11Li alloy during the extrusion process shows up as greatly refining the DRXed grains. The original intermetallic phases induced by Y/Er and Zn addition are crushed into relatively fine particles during extrusion, which can promote DRX by the PSN mechanism.

(3) The addition of Y/Er and Zn can remarkably improve room temperature strength of the as-extruded Mg-11Li alloy. This is mainly ascribed to the refined DRXed grains and numerous dispersed intermetallic phase particles.

## Figures and Tables

**Table 1:** Chemical composition of the investigated alloys (wt %).

Alloy	Li	Y	Er	Zn	Mg
Mg-11Li	11.65	–	–	–	Bal.
Mg-11Li-4Y-2Er-2Zn	11.61	4.32	1.83	2.11	Bal.
Mg-11Li-8Y-4Er-4Zn	11.75	7.74	4.32	3.53	Bal.

**Table 2:** Average tensile properties including ultimate tensile strength (UTS), yield strength (YS), and *ε* of the studied alloys.

Samples	*ρ* (g/cm^3^)	UTS (MPa)	YS (MPa)	*ε* (%)
Mg-11Li	1.394	145 ± 2	117 ± 3	22 ± 2
Mg-11Li-4Y-2Er-2Zn	1.459	224 ± 3	174 ± 3	16 ± 2
Mg-11Li-8Y-4Er-4Zn	1.530	243 ± 3	210 ± 3	15 ± 2
